# The Elevational Distribution Patterns of Plant Diversity and Phylogenetic Structure Vary Geographically Across Eight Subtropical Mountains

**DOI:** 10.1002/ece3.70722

**Published:** 2024-12-17

**Authors:** Kuiling Zu, Fusheng Chen, Chao Huang, Yuanqiu Liu, Fangchao Wang, Guojin Zhu, Wensheng Bu, Xiangmin Fang, Liping Guo

**Affiliations:** ^1^ Jiangxi Key Laboratory of Subtropical Forest Resources Cultivation and Grassland Administration on Forest Ecosystem Protection and Restoration of Poyang Lake Watershed, College of Forestry Jiangxi Agricultural University Nanchang China; ^2^ Jiangxi Provincial Key Laboratory of Conservation Biology Jiangxi Agricultural University Nanchang China

**Keywords:** biodiversity conservation, elevational diversity patterns, evolutional history, non‐native species, phylogenetic structure, species richness

## Abstract

Mountains have been recognized as biodiversity hotspots and possess strong elevational gradients. Whether these gradients exhibit similar characteristics in the multidimensional diversity patterns across different mountain ranges is a subject of inquiry. Exploring the elevational patterns of the diversity and phylogenetic information of plant species in the different subtropical mountains is necessary. Here, we compiled the elevational patterns of plant diversity occurring in the eight subtropical mountains of China and focused on the assessment of the patterns and determinants of the multi‐dimensional diversity and phylogenetic structure in different mountains. We also detected the elevational patterns and their relationship between different groups. The results indicate two main patterns of multi‐dimensional diversity: monotonic decrease and hump‐shaped, along with the area effect on the species diversity. There is a strong positive link between the non‐native and native species of species richness, and significant differences in phylogenetic structure's elevational distribution. We did not find the same rule in the mountains that the plant species in the lowlands indicate phylogenetic overdispersion, and the species in the higher elevation regions indicate phylogenetic clustering. We found that the plants' diversity peak is related to the mountains size, and this result showed that we should pay more attention to the conservation of plant communities in the higher elevation regions for the higher mountains. This study suggested that we should take different protective measures for the subtropical mountains: the lowland regions should be paid more attention in Mt. Lushan (LS), Mt. Guanshan (GS), Mt. Huanggang (HG), and Mt. Fanjing (FJ), and the middle‐altitude regions should be of concern for other mountains. This study helps to better understand the elevational gradients of species diversity on different scales and provides supporting scientific basis for biodiversity conservation in the subtropical mountain regions.

## Graphical abstract

Both monotonic decrease and hump‐shaped patterns exist in the subtropical mountains. The regions with more native plant species where the non‐native species tend to be more. There is no uniform rule that the plant species in lowlands indicate phylogenetic overdispersion. The plants' diversity peak is related to the relative elevation of the mountains.

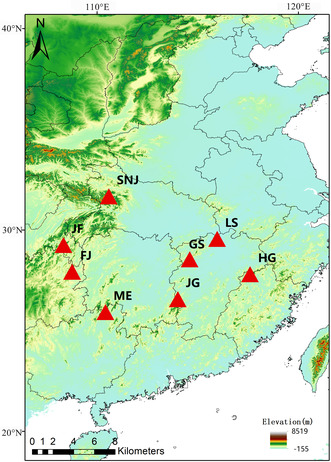

## 1 Introduction

Biodiversity represents the variety of all living species on Earth, while ongoing climate warming and the pressure of anthropogenic activities lead to biodiversity losses in different degrees (Auffret and Svenning 2022; Boonman et al. 2024; Wiens and Zelinka 2024; Zheng and Cao 2015). The patterns of species diversity and distributions in different regions are importance to the understanding for the biodiversity loss. Biodiversity distribution will also help to better understand the framework targets for the biodiversity conservation and ecological restoration (Sacco et al. 2021). Consequently, understanding the plant diversity and distribution provides important insights for effective biodiversity conservation and ecological restoration.

Many mountainous areas are known as biodiversity hotspots, and mountains are of great importance in the biodiversity conservation (Bomhard and Bertzky 2010; McCain and Grytnes 2010; Thakur et al. 2022; Zu et al. 2024). A handful of great scientists such as Darwin, Wallace, and von Humboldt have conducted scientific investigations and observations in the mountainous regions, and the distribution of species diversity across the elevational gradients were found (Lomolino, 2001; McCain and Grytnes 2010). Previous studies on the elevational patterns have indicated the presence of four distinct types of decreasing diversity: the low plateau, a low plateau with a mid‐peak, and a unimodal mid‐elevation peak within mountainous regions (McCain 2009). However, it is controversial whether there is consistent regularity of species diversity along the elevational gradients in subtropical mountain regions. Besides, with the impact of climate change and human activities (including tourism and road development), more and more non‐native species colonize in the mountain regions (Tecco et al. 2016). The spread of non‐native species will pose a serious threat to forest ecosystem in mountains (Pathak et al. 2021; Mcdougall et al. 2011). Understanding the distribution pattern of non‐native species and its relationship with native species diversity in subtropical mountain will provide basic information for the study of biodiversity conservation and biodiversity changes in mountainous regions.

Mountains have strong elevational gradients in the climatic conditions and vegetation coversa, which makes it an ideal “natural laboratory” to study the elevational distribution pattern of plant diversity. Indeed, species richness (SR) across the elevational gradients has been reported in the previous studies (Beck et al. 2016; He et al. 2019; Lee, Chun, and Ahn 2013). Species with different evolutionary histories contribute differently to the process of community assembly, and studies of the plant diversity that rely only on taxonomic diversity may ignore the unique evolutionary history of taxonomic groups (Daru et al. 2014; Mouillot et al. 2016). Therefore, only using this index alone to assess biodiversity cannot fully reflect the diversity characteristics of the study regions. However, the species’ multi‐dimensional diversity including phylogenetic diversity (PD) and phylogenetic endemism (PE), offers a more comprehensive understanding of their evolutionary history. This, in turn, plays a crucial role in the conservation of biodiversity within mountainous regions (Faith and Baker 2006; Thuiller et al. 2014). Phylogenetic diversity (PD) reflects the accumulation of evolutionary history of taxa in the study regions. For the same species richness, higher PD means longer branches length, which indicated that species in a community accumulate more evolutionary time and have more recent‐originated species (Tucker et al. 2019). Conversely, low phylogenetic diversity corresponds to younger groups or closely related groups. This index has been widely used in the diversity research (Wang et al. 2023; Zhou et al. 2020). The phylogenetic endemic (PE) combines phylogenetic information and species distribution range, and has been regarded as another useful index helping to explore the biodiversity conservation gap from the perspective of the uniqueness of phylogenetic evolutionary history (Guerin and Lowe 2015; Rosauer et al. 2009). Therefore, the incorporation of both taxonomic and phylogenetic diversity should be used in conservation practice, which is used to comprehensively compare and integrate the results of different indicators into biodiversity conservation. Besides, with the acceleration of globalization, biological invasions have become common phenomenon, and the non‐native species might be influence the distribution of the native species (Seebens et al. 2017). Understanding the elevational patterns of the native and non‐native plant species is useful for the disturbance of alien species to mountain ecosystem.

Using evolutionary relationships of DNA sequences to represent phylogenetic relationships between species could not only more effectively measure community species composition, but also help to understand the phylogenetic structure of the different community (Webb et al. 2002). Previous study has reported that closely related species exhibit a striking similarity in their functional traits, and those species demonstrate a greater consistency in their adaptive capabilities to similar environments (Prinzing et al. 2001). If habitat filtering plays an important role in the region or community, closely related species will be selectively excluded within the same habitat or environment (Wiens and Graham 2005). Conversely, competitive exclusion makes it impossible for species with similar ecological niches to coexist in the same environment, so species in the region or community are less closely related. Therefore, research on phylogenetic structure combining evolution and ecology could deeply analyze the causes of community species composition from an evolutionary perspective and help improve macro‐ecological theory. However, a recent study on the phylogenetic structure of plants on mountain elevation gradients showed that there are still lacking attention about the phylogenetic structure of plants species in the subtropical mountains (Qian, Ricklefs, and Thuiller 2021). Filling this geographical gap of subtropical mountains are helpful to understand the truly patterns of the phylogenetic structure in mountain ecosystems across the world. In addition, whether the elevational distribution of phylogenetic structure in different mountains of subtropical regions is consistent, and this question is important for understanding the history of plant assembly among different regions.

Many previous studies of the taxonomic and phylogenetic diversity along the elevational gradients were based on the single research sites such as Taibai Mountain in China (Xu et al. 2021), Namjagbarwa mountains (Sun et al. 2020), and central Himalayas (Liang et al. 2020). Few studies have been conducted to detect the similarities and differences in species diversity patterns in different mountains of subtropical regions (Gheyret et al. 2020; Guo et al. 2013). The subtropical region of China is vast and rich in biodiversity; for instance, the species diversity of forests in the subtropical regions is higher than that in the same latitude of North America (Song 2013). The evergreen broad‐leaved forest is the zonal vegetation of subtropical forest ecosystem in China and the main body of subtropical forest biodiversity. However, the species composition and diversity of the evergreen broad‐leaved forest communities may vary due to differences in the location and elevation of different subtropical mountains. This study selected eight subtropical mountains to compare the distribution of species diversity and phylogenetic structure. This study aims to detect the following four issues: (1) What is the elevational patterns of plant multi‐dimensional diversity in subtropical mountains? (2) Whether the elevational patterns of the native and non‐native plant species is consistent? (3) What is the phylogenetic structure along the elevational gradient in the subtropical mountains? and (4) What determines the peak of plant richness in the subtropical mountains? The study provides a theoretical basis and suggestions for biodiversity conservation in subtropical mountains.

## 2 Material and Methods

### 2.1 Study Area

**FIGURE 1:**
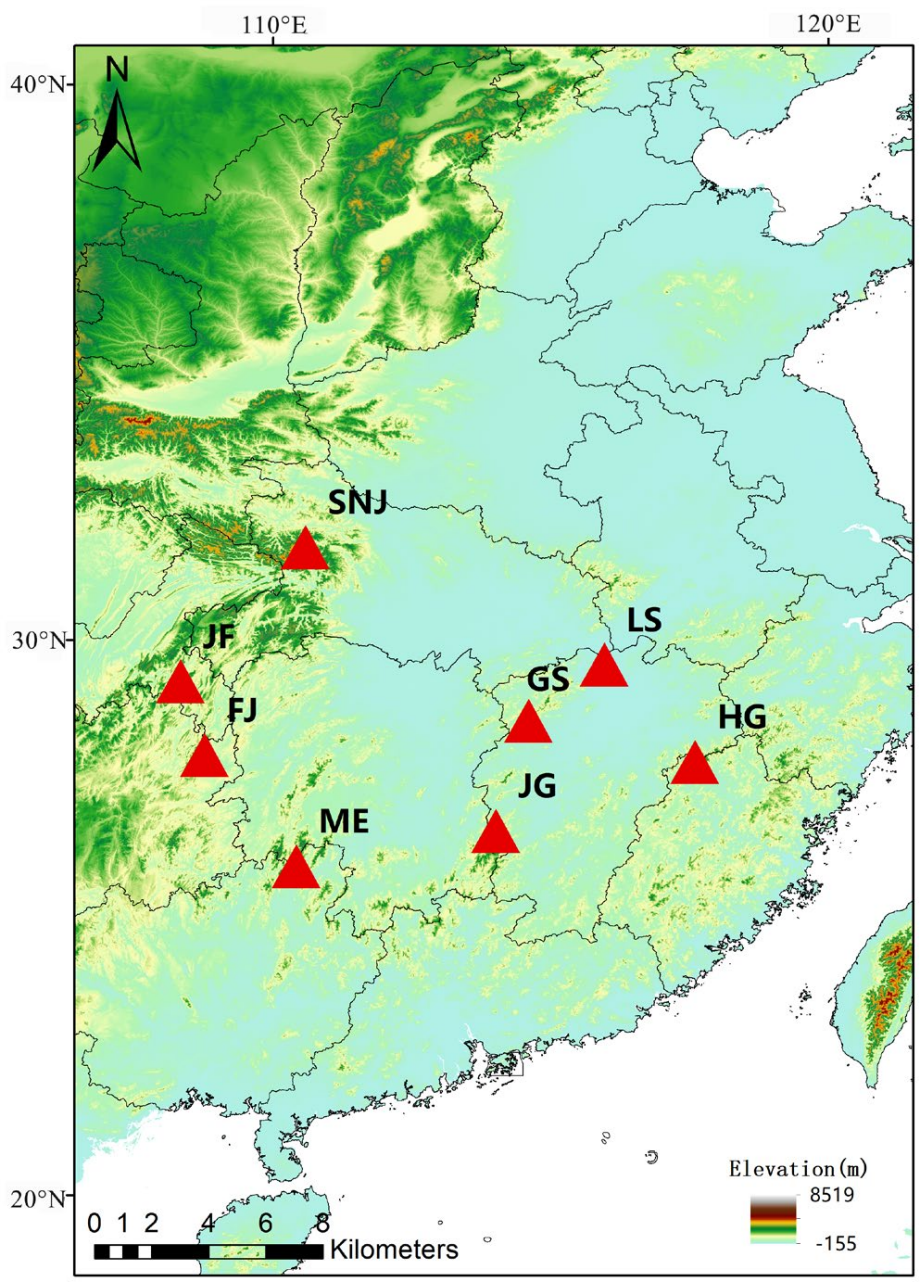
The study mountains located in the subtropical regions of China. The red triangle represents each mountain. GS, Mt. Guanshan; LS, Mt. Lushan; JG, Mt. Jinggang; SN, Mt. Shennong; FJ, Mt. Fanjing; HG, Mt. Huanggang; JF, Mt. Jinfo; and ME, Mt. Maoer.

Eight representative mountains located in the subtropical regions of China were selected, and these mountain sites are belong to eight distinct mountain ranges. The eight mountain ranges are Jiuling, Mufu, Wuling, Wuyi, Daba, Dalou, Luoxiao, and Nanling. Among them, Nanling mountain ranges have the most intact subtropical evergreen broad‐leaved forest in the belt dominated by deserts at the same latitude on Earth, and it is a key region for biodiversity conservation. So we select one typical mountain site on each mountain ranges. The mountain sites are Mt. Guanshan, Mt. Lushan, Mt. Jinggang (JG), Mt. Shennong (SN), Mt. Fanjing, Mt. Huanggang, Mt. Jinfo (JF), and Mt. Maoer (ME) (Table 1 and Figure 1). Those mountains have higher elevation gradients and obvious differentiation of vegetation types. These mountain sites have distinctive elevational distribution of plant diversity, providing a comprehensive representation of subtropical mountainous. The mountains are distributed across five provinces of Jiangxi, Guizhou, Hubei, Chongqing, and Guangxi. The tallest mountain is Mt. Shennong, and the highest altitude is 3106 m and the altitude range is about 2706 m. The lower mountains are the Mt. Lushan and Mt. Guanshan that located in the north and central of Jiangxi province, and their highest altitudes are 1474 and 1480 m, respectively. Mt. Guanshan has the warm and humid southeastern monsoon climate zone of the central subtropics, and its altitude range is about 1250 m. Mt. Huanggang is the highest mountain of Wuyi mountain ranges, and its altitude range is about 1808 m. Mt. Maoer is the highest mountain of Nanling mountain ranges, and its altitude range is about 1862 m. These two sites are well preserved in the natural evergreen broad‐leaved forest and representative the subtropical regions of China.

**TABLE 1:** Location of the eight study regions.

Mountain range	Mount	Abbr.	Elevation	Latitude	Longitude	Province	Data
Jiuling range	Guanshan	GS	1480	28.54	114.60569	Jiangxi	10,800
Mufu range	Lushan	LS	1474	29.55	115.967	Jiangxi	19,261
Wuling range	Fanjing	FJ	2493	27.917	108.767	Guizhou	7146
Wuyi range	Huanggang	HG	2158	27.8	117.6	Jiangxi	11,821
Daba range	Shennongjia	SN	3106	31.667	110.583	Hubei	59,131
Dalou range	Jinfo	JF	2251	29.25	108.333	Chongqing	40,841
Luoxiao range	Jinggang	JG	2120	26.558	114.01667	Jiangxi	22,041
Nanling range	Maoer	ME	2142	25.918	110.42	Guangxi	7105

*Note:* Mount means the study site. Abbr. = abbreviation, Elevation = The highest elevation, “data” means the total amount of data after cleaning the distribution data at different mountains. GS, Mt. Guanshan; LS, Mt. Lushan; JG, Mt. Jinggang; SN, Mt. Shennong; FJ, Mt. Fanjing; HG, Mt. Huanggang; JF, Mt. Jinfo; and ME, Mt. Maoer.

### 2.2 Data Sources of Species Diversity

The plant distribution information was obtained from the historical specimens collected from museum and herbarium collections, original field surveys in some mountains in these years, and the literature and other scientific research books (Table S1). Herbarium records were mainly extracted from the National Specimen Information Infrastructure (NSII, http://www.nsii.org.cn) and the Chinese Virtual Herbarium (CVH, http://www.cvh.ac.cn). In addition, the original botanical surveys were conducted in Mt. Lushan, Mt. Guanshan, Mt. Jinfoshan, and Mt. Jinggang. We carried out botanical survey by setting the vegetation plots of 20 × 30 m along the elevational gradients. The plots along elevational gradients were established by 50 m interval from low to high elevations. In addition, to ensure the data integrity, we also obtained plant distribution data points during the past years from literature and books (Table S1). The main reference books are Chinese evergreen broad‐leaved forest (Song 2013), Comprehensive Scientific Expedition on Biodiversity in Jinggang Mountains, China (Liao et al. 2014), and Investigation and study on biodiversity in Lushan Nature Reserve of Jiangxi Province (Liu and Wang 2010).

### 2.3 Plant Diversity Data Cleaning

To ensure the data reliability and quality, we did a rigorous cleaning of the plant species' distribution data. Firstly, we only include plant specimens with accurate altitude, removing duplicate records and plant specimens lacking precise geographic information. Secondly, all recorded plant species names have been standardized according to the Catalogue of Life (COL, http://www.catalogueoflife.org/), the plant list (TPL, http://www.theplantlist.org/) as well as the Taxonomic Name Resolution Service (TNRS, http://tnrs.iplantcollaborative.org/). We also calibrated the family and genus for each plant species based on the Flora of China (FOC, https://www.iplant.cn). The data merging and cleaning were processed in R 3.3.3 (R script, https://doi.org/10.17632/pghkfm5sm9.3). The total amount of data about herbarium specimens we choose in each mountain can be found in Table 1.

### 2.4 Calculation of Plant Diversity Index

We calculated the diversity indices for each mountain along the elevational gradients, which were including species richness, phylogenetic diversity, and phylogenetic endemism in our study. For each mountains, we divided the elevation gradients into different elevational belts at 100 m vertical intervals. This method is widely used in the study of spatial patterns of phylogenetic structure for ferns, non‐native and native plant species along the elevational gradient (Qian, Kessler, and Jin 2022). Then, we calculated each of the three diversity indices of elevational belts for each mountain. In particular, species richness is the universal index which is widely used in the studies about biodiversity or the patterns of elevation and latitude (Wang et al. 2011). Faith's PD was an index which could be used to estimate the phylogenetic diversity of plant species within each regions or elevational belts (Faith and Baker 2006). Phylogenetic endemism (PE) included the evolutionary and spatial information and could be capable of measuring the range of the species distribution and its range‐constrained phylogenetic diversity (Rosauer et al. 2009; Zu et al. 2023). In order to calculate the phylogenetic diversity and phylogenetic endemism for each mountain, we should get the phylogenetic tree of the plant species for each mountain. Also, Jaccard similarity index (*β*_*j*_) and Cody substitution index (*β*_*c*_) were used to estimate the β‐diversity for the eight mountains (Tang et al. 2013).

The calculation formula of β‐diversity is as follows:

(1) $$\;\beta_{j}=\frac{c}{\;a+b+c}$$

(2) $$\;\beta_{c}=\frac{\;a+b-2c}{c}$$

Among that, a and b are the number of species of the two communities, respectively, and c is the number of common species of the two communities.

We first constructed the phylogenetic tree using the R package “V. PhyloMaker2,” “picante,” “ape,” “phyloregion,” and the phylo.maker function under scenario 1 was used in this study (Jin and Qian 2022). Then, we used a phylogenetic tree containing a large number of vascular plants to calculate the above three diversity indices (Jin and Qian 2022). Based on this big tree of all the vascular plants, we calculated the phylogenetic diversity and phylogenetic endemism along the elevational gradients for each mountain. We built a tree for each mountain and then calculated the diversity indices.

### 2.5 Phylogenetic Structure Along the Elevational Gradients

The net relatedness index (NRI) was used to represent the phylogenetic structure, and we quantified the NRI values along the elevational belts of each mountain. The NRI values were calculated by the Mean Phylogenetic Distance (MPD) and the average phylogenetic distance between all pairs of the species in the different mountains (Webb et al. 2002).

The calculation formula of NRI index is as follows:

(3) $$\mathrm{NRI}=-1\times \left({\mathrm{MPD}}_{o}-{\mathrm{MPD}}_{r}\right)/\mathrm{sd}\;\left({\mathrm{MPD}}_{r}\right)$$

where MPD_o_ represents the actual observed mean phylogenetic distance, MPD_r_ represents the average distance of species randomly placed on the phylogenetic tree, and sd (MPD_r_) are standard deviation of MPD_r_. We built a null model to calculated MPD_r_ of 1000 randomly generated communities from a given species pool based on the phylogenetic tree.

An NRI greater than zero suggests phylogenetic clustering, indicating that coexisting species tend to be more closely related to each other than expected. Conversely, an NRI less than zero suggests phylogenetic overdispersion, indicating that coexisting species tend to be less closely related than expected (Webb et al. 2002). We calculated the NRI for each elevational belt in different mountains using “Ape” and “Picante” R packages (R Development Core Team 2017).

### 2.6 Data Analysis

In order to detect whether the elevational patterns of the native and non‐native plant species are consistent, we get the distribution of non‐native plant species. Distinguishing non‐native plant species mainly based on the dataset of catalog of alien plants in China (Lin et al. 2022). We examine the distribution patterns of three diversity indices for both native and non‐native plants across eight subtropical mountains. Then, we calculated the non‐native species numbers and elevational distributions in each mountain, and then identify the relationship between the species richness of native and non‐native species. In order to understand detected the area and mid‐domain effect hypothesis explaining the distributional elevation pattern, we calculated the area of different elevational belts for the eight mountains based on the ArcGis 10.2 software.

In order to understand what determines the peak of plant richness in the subtropical mountains, we evaluated the determinants of each mountain's highest elevation distribution points, mainly from the perspective of mountain characteristics (relative elevation, location) and climate factors (temperature, precipitation). In particular, the relative elevation of each mountain was according to the distribution range of each mountain and also combined with the elevation map (SDM, 1km^2^). Then, we obtain the lowest and highest elevation of each mountain based on the ArcGis 10.2 software. Similarly, the location of each mountain was the center point (latitude and longitude) of the distribution range of each mountain. The climate factors include the annual mean temperature (AMT) and annual precipitation (AP) for each mountain, and the climate data were extracted from the WorldClim database (http://www.worldclim.org) at the spatial resolution of 30‐arc‐second (1km^2^). ArcGis 10.2 software was used to extract the annual mean temperature and annual precipitation above for each mountain. The generalized linear models (GLMs) with quasi‐Poisson residuals were used to identify the relationship between the peak of plant richness and the factors, and the slopes and the explanatory power of each variable (*R* ^2^) were extracted. We also evaluated the elevational distribution pattern of phylogenetic structure in different mountains. In order to detect the significant relationships between the phylogenetic structure and elevation in these mountains, we selected the median of each elevational belts as the X‐axis along the mountain elevational gradients for each mountain site. GLMs were used to detect the significant relationships between the phylogenetic structure and elevation in these mountains, the correlation between the phylogenetic structure, and the number of species in different mountains.

## 3 Result

### 3.1 Elevational Patterns of the Taxonomic and Phylogenetic Diversity in the Subtropics Mountains

**FIGURE 2:**
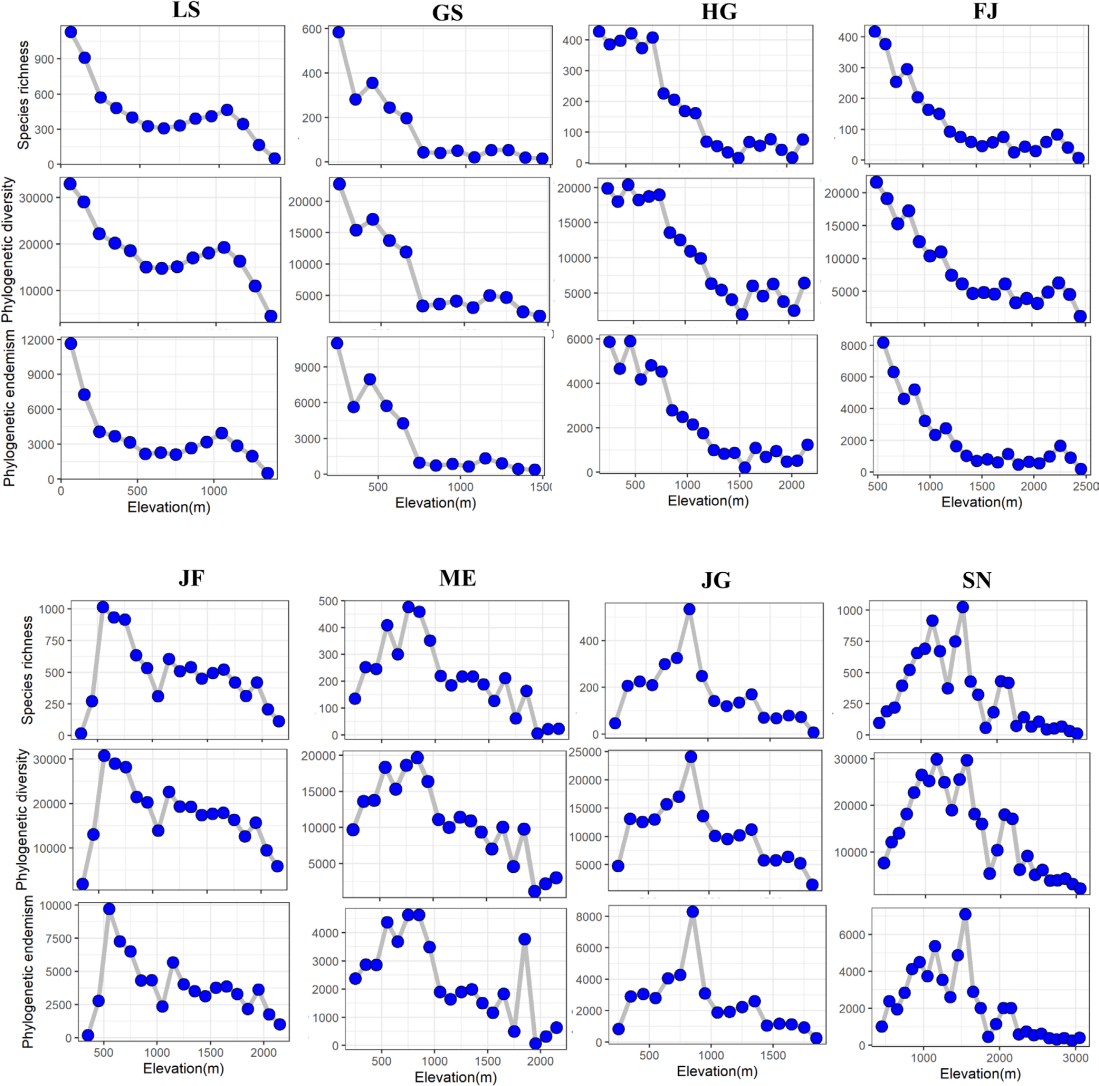
The distribution patterns in species richness, phylogenetic diversity, and phylogenetic endemism across the elevational gradients of the eight selected mountains. GS, Mt. Guanshan; LS, Mt. Lushan; JG, Mt. Jinggang; SN, Mt. Shennong; FJ, Mt. Fanjing; HG, Mt. Huanggang; JF, Mt. Jinfo; and ME, Mt. Maoer.

**FIGURE 3:**
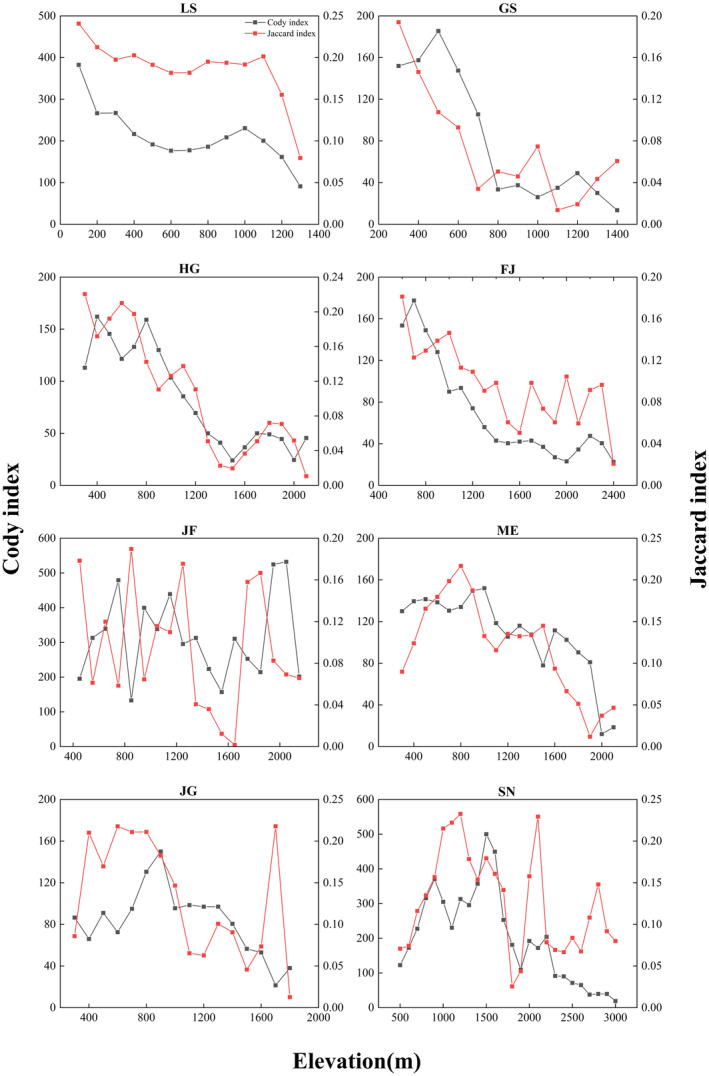
Jaccard similarity and Cody index between plant communities at adjacent elevation gradients. GS, Mt. Guanshan; LS, Mt. Lushan; JG, Mt. Jinggang; SN, Mt. Shennong; FJ, Mt. Fanjing; HG, Mt. Huanggang; JF, Mt. Jinfo; and ME, Mt. Maoer.

**FIGURE 4:**
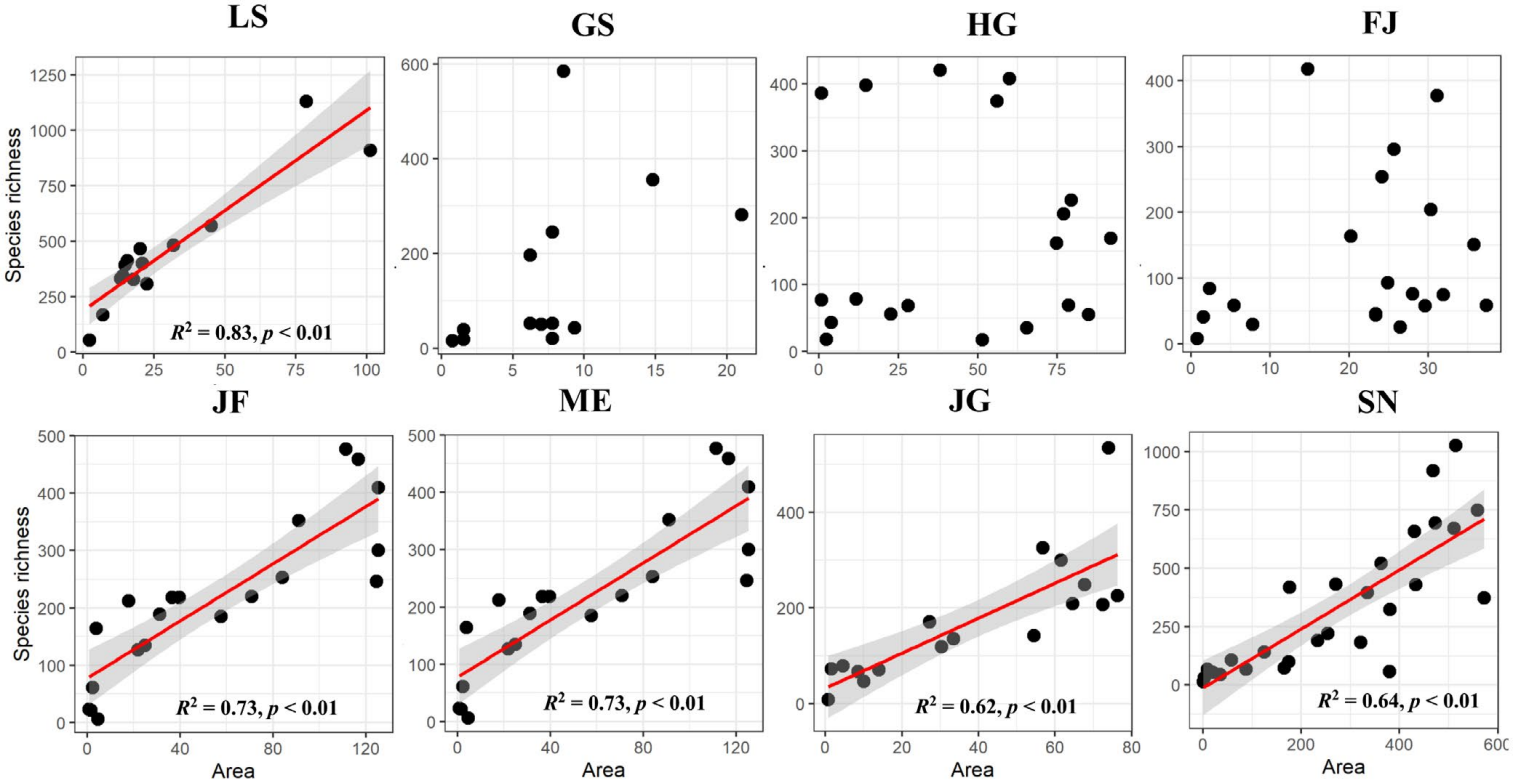
Relationships between the species richness and the area of different mountains. GS, Mt. Guanshan; LS, Mt. Lushan; JG, Mt. Jinggang; SN, Mt. Shennong; FJ, Mt. Fanjing; HG, Mt. Huanggang; JF, Mt. Jinfo; and ME, Mt. Maoer.

Species richness of the eight mountains ranged from 1326 to 3486 species per mountains, and the mean numbers were the 1978 species. The number of genera across eight mountains ranged from 588 to 947, with an average of 738 genus per mountain. The number of families across the eight mountains ranged from 164 to 188, with an average of 173 families per mountain (Table 2). The three diversity index of species richness, phylogenetic diversity, and phylogenetic endemism had similar distribution patterns along the elevational gradients in the eight mountains (Figure 2). Both Jaccard similarity index (*β*_*j*_) and Cody substitution index (*β*_*c*_) showed a decreasing trend with the elevation increase, and *β*_*j*_ is lower at high altitudes except Mt. Jinfo and Mt. Jingang (Figure 3). Those elevational distribution patterns are mainly two types: monotonic decrease and hump‐shaped patterns. Concretely speaking, both the taxonomic and phylogenetic diversity showed a monotonic descending pattern in Mt. Lushan, Mt. Guanshan, Mt. Huanggang, and Mt. Fanjing (Figure 2). That is to say, the multi‐dimensional diversity was highest in the lower elevations of these mountains and suggested that the lowland should be taken more attention of the biodiversity conservation. Both the taxonomic and phylogenetic diversity showed a hump‐shaped pattern in Mt. Jinfo, Mt. Maoer, Mt. Jianggang, and Mt. Shennong along elevational gradients (Figure 2). However, the highest elevations at which the species richness of the four mountains occurs are different. The peaks of Mt. Jinfo occur between 500 and 600 m, Mt. Maoer occurs between 700 and 800 m, Mt. Jianggang occurs between 800 and 900 m, and Mt. Shennong occurs between 1500 and 1600 m a.s.l. For the “hump‐shape distributional patterns” mountains, the area of elevational pattern shows a hump‐shape distributional pattern along the elevation, indicating that the area in the middle‐elevation regions is the largest (Figure S1). There is one “monotonic decrease” mountain that has a significant positive correlation between the species richness and area. For the four “hump‐shape distributional patterns” mountains, there is significant positive correlation between the species richness and area of different elevational belts (Figure 4).

**TABLE 2:** Numbers of seed plants' species, genus and family in different mountains.

Mount	Abbr.	Species	Genus	Family
Guanshan	GS	1371	623	174
Lushan	LS	2068	882	188
Fanjing	FJ	1386	588	164
Huanggang	HG	1768	718	177
Shennongjia	SN	2575	851	171
Jinfo	JF	3486	947	180
Jinggang	JG	1326	609	168
Maoer	ME	1840	684	164

### 3.2 Relationships Between the Native and Non‐native of Species Richness Along the Elevational Gradients

**FIGURE 5:**
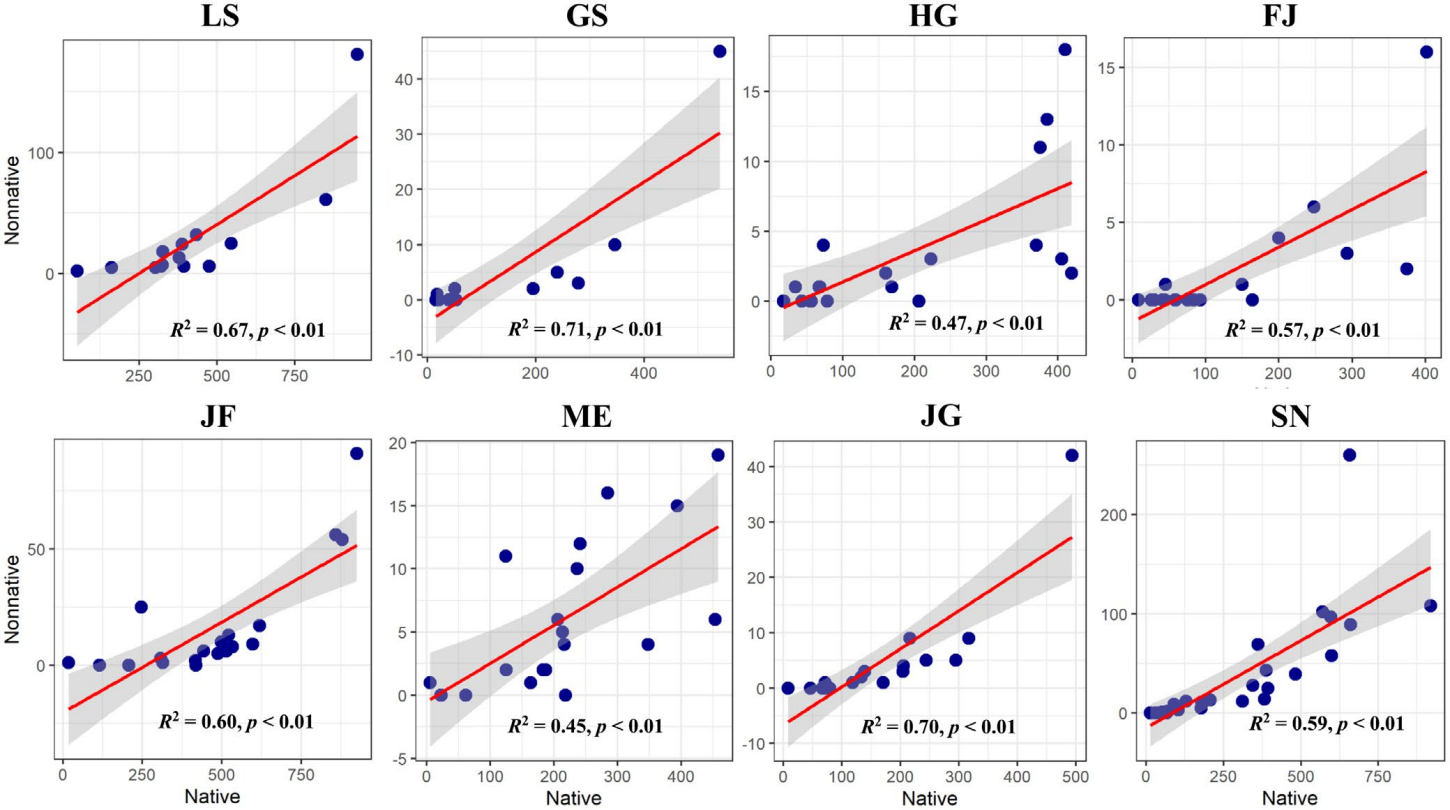
Relationships between non‐native and native species of species richness along the elevational gradients. GS, Mt. Guanshan; LS, Mt. Lushan; JG, Mt. Jinggang; SN, Mt. Shennong; FJ, Mt. Fanjing; HG, Mt. Huanggang; JF, Mt. Jinfo; and ME, Mt. Maoer.

In this study, the species diversity of non‐native species of the eight mountains ranged from 26 to 207 species per site. The number of genus of the eight mountains ranged from 26 to 153 species, and the number of families of the eight mountains ranged from 16 to 62 species per study sites. Mt. Lushan, Mt. Jinfo, and Mt. Shennong have more non‐native species (Table S2). For the eight mountains in the subtropics region, a strong and significant positive correlation was found between the non‐native and native species of the species richness across the elevational gradients (Figure 5). This result suggests that the species richness showed similar patterns in both the non‐native and native species in the eight mountains, and the regions with more native species where the non‐native species tend to be more.

### 3.3 Elevational Patterns of Phylogenetic Structure in the Subtropics Mountains

**FIGURE 6:**
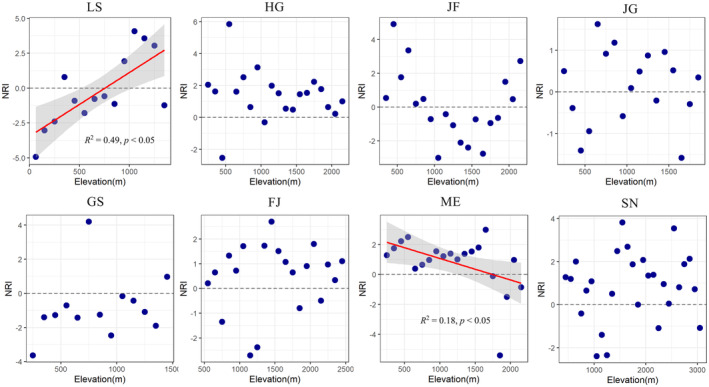
The net relatedness index (NRI) of plant species along the elevational gradients in the eight mountains. GS, Mt. Guanshan; LS, Mt. Lushan; JG, Mt. Jinggang; SN, Mt. Shennong; FJ, Mt. Fanjing; HG, Mt. Huanggang; JF, Mt. Jinfo; and ME, Mt. Maoer.

In LS, NRI showed a decreasing pattern along the elevational gradients (*R* ^2^ = 0.49, *p* < 0.05). In contrast, NRI showed an increasing pattern across the elevational gradients (*R* ^2^ = 0.18, *p* < 0.05) in Mt. Maoer. This result suggested that the phylogenetic clustering of the plant species occurs in the lower elevational regions, while phylogenetic overdispersion occurs in the higher elevational regions (Figure 6). However, it showed no significant correlation with the elevation in other mountains (HG, JF, JG, GS, FJ, SN). The HG, JF, FJ, and SN plant species tend to be more phylogenetically clustered at lower and higher elevations. However, the species in GS tend to be phylogenetically overdispersed in most of the elevational belts.

### 3.4 Determinants of the Plants' Diversity Peak and Phylogenetic Structure

**FIGURE 7:**
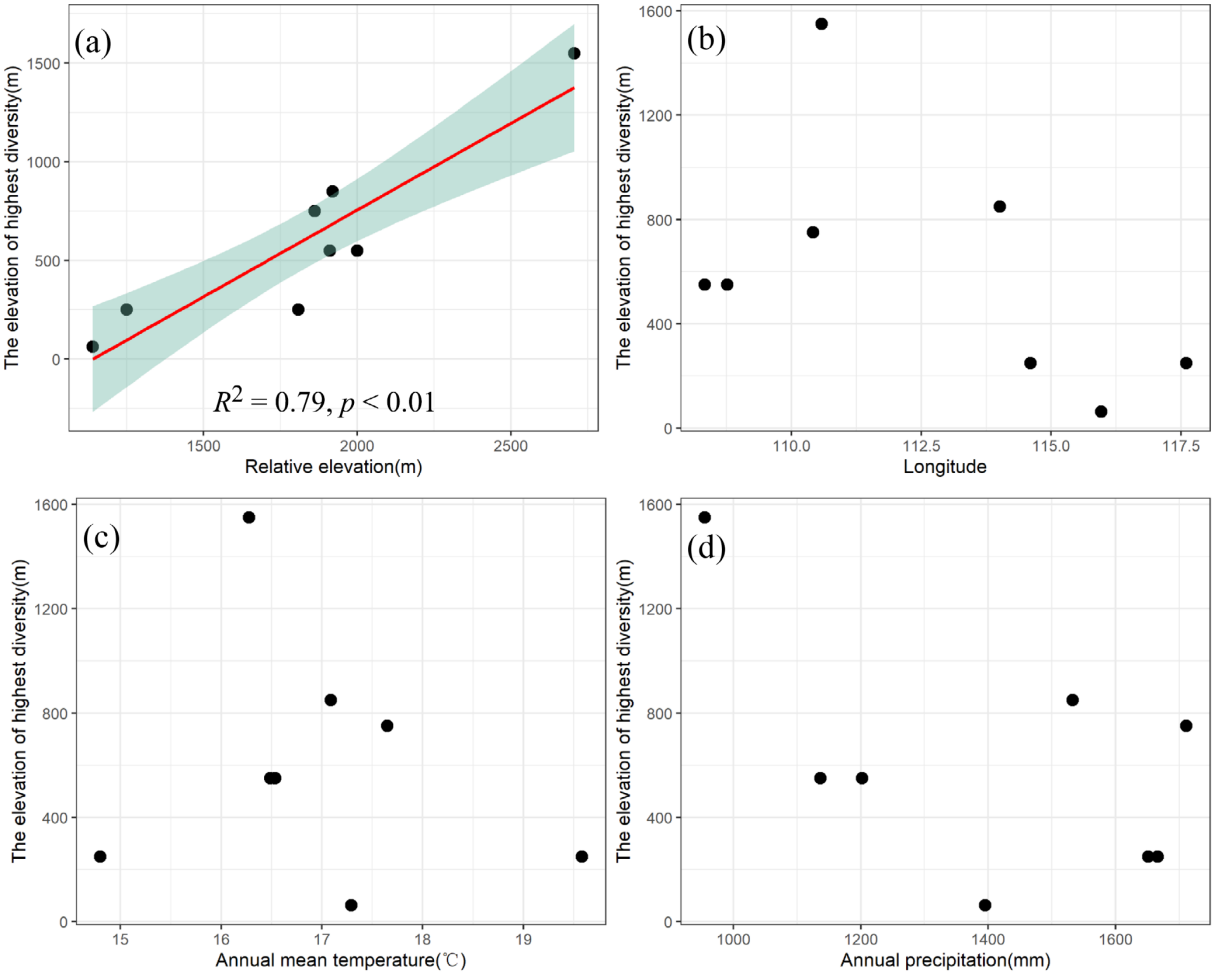
The relationship between the plants' diversity peak and relative elevation (a) the longitude (b) annual mean temperature (c) and annual precipitation (d) of the subtropical mountains.

The relative elevation positively correlated with the plants' diversity peak (*R* ^2^ = 0.79, *p* < 0.01). However, the plants' diversity peak had no significant correlations with the location of the mountains (longitude), the annual mean temperature, and precipitation of the mountains (Figure 7). Our result shows a significant negative relationship between the phylogenetic structure and the species numbers in Mt. Lushan. It indicated that the community with more species and the phylogenetic structure of the species tend to be over dispersed. However, we did not find a significant relationship between the phylogenetic structure and the species numbers in some other mountains (Table 3). The phylogenetic structure is unrelated to the number of species in most of the subtropical mountains.

**TABLE 3:** The relationship between the phylogenetic structure and the species numbers of different mountains.

Abbr.	Slope	*R* ^2^	*p*
LS	−0.01	0.31	0.04
GS	−0.01	0.24	0.09
HG	0.00	0.06	0.32
FJ	0.00	0.02	0.56
JF	0.00	0.00	0.84
ME	0.00	0.11	0.16
JG	0.00	0.06	0.36
SN	0.00	0.00	0.77

*Note:* GS, Mt. Guanshan; LS, Mt. Lushan; JG, Mt. Jinggang; SN, Mt. Shennong; FJ, Mt. Fanjing; HG, Mt. Huanggang; JF, Mt. Jinfo; and ME, Mt. Maoer.

## 4 Discussion

### 4.1 Regional Difference in the Species Diversity Across the Elevational Gradients

Our results show that subtropical mountains exhibit varied elevational diversity patterns, with species richness and phylogenetic diversity following either monotonic decrease or hump‐shaped patterns along gradients, aligning with Rahbek's (2005) review that identifies the hump‐shaped pattern as most typical, succeeded by the monotonic decrease pattern. Previous studies also suggested that subtropical forest biomass, productivity, and biodiversity decline with the increase in altitude (Unger, Leuschner, and Homeier 2010). Similar to this result, recent study has found that the tree species richness of both mountains (Mt. Wuyi and Mt. Gongga) decreased with the increase in elevation (Cai et al. 2023). In addition to the monotone decreasing pattern, this study also found that the elevation gradient of plant diversity in other four subtropical mountains showed a hump‐shaped patterns. Our result insisted the area and mid‐domain effect hypothesis, and the water–energy balance hypothesis (Colwell, Rahbek, and Gotelli 2004; O'Brien 2006). The hump‐shaped patterns are consistent with those observed in previous studies for the seed plants elevational distribution in Mt. Gongga (Zu et al. 2019), Namjagbarwa mountains (Sun et al. 2020), central Himalayas (Liang et al. 2020), and Changbai mountains (Qian, Hao, and Zhang 2014). The previous study indicated that mountains with greater elevational extent were more likely to display the hump‐shape distributional patterns (Guo et al. 2013). However, our results do not support the conclusion that higher elevations extent can easily show the hump‐shape distributional patterns. For example, Mt. Fanjing (2000 m) and Mt. Huanggang (1808 m) have a higher relative altitude, and their relative elevations are similar to Mt. Jinfo and Mt. Maoer. However, Mt. Fanjing and Mt. Huanggang show a monotonically decreasing distribution pattern, which is different from Mt. Jinfo and Mt. Maoer. The distribution patterns in Mt. Fanjing and Mt. Huanggang are related to their relative altitude and may be cause by a combination of multiple environmental factors. Meanwhile, we should not only focus on these distribution patterns, but also on regions with higher biodiversity along different mountain elevation gradients to provide more conservation advice.

In this study, we found that the regions with more non‐native species were the regions with more native plant species, which is not due to the failure of the biodiversity barrier mechanism, but may be because the regions with rich biodiversity tend to have better resource availability. This result differs from a recent study of species richness patterns of alien and native plants along an elevational gradient in the Himalayas, which concluded that the elevational patterns of alien and native species were different (Manish 2021). Our results support the resource opportunity hypothesis, which states that available resources are the key factors determining the invasiveness of ecosystems. Once a community has the necessary ecological resources for non‐native species, the community will be able to obtain the necessary ecological resources. In addition, most of these ecological resources have not been effectively used by native species, that is, there are empty ecological niches and biological invasions will occur as soon as the propagules of alien species enter (Davis, Grime, and Thompson 2000).

Moreover, our results also show that in the subtropical region, the difference in elevational distribution patterns of plant diversity is not significantly correlated with the location of the mountain and the climatic factors (temperature and precipitation), but is positively correlated with its mountain characteristics, such as the relative altitude of the mountain. The results suggest that the higher the relative altitude of the mountain, the higher the elevational distribution of its diversity. The reason may be that the higher the elevation of the diversity peaks, the higher the elevation of the mountains with more samples (Guo et al. 2013). However, we did not find that the peak of species diversity was related to climate factors, which may be caused by the low sample size selected in this study. We will further evaluate the influence of peak of plant diversity based on meta‐analysis.

### 4.2 Spatial Differentiation of Phylogenetic Structure Along the Elevational Gradients

This study did not find a uniform rule to indicate that a habitat must correspond to a particular phylogenetic structure. The results of the patterns of NRI along the elevation were even reversed in different mountains. For instance, NRI has a significant positive correlation with the elevation in Mt. Lushan, and the result showed that plant species at higher elevations are more phylogenetically clustered. Conversely, NRI has a significant negative correlation with the elevation in Mt. Maoer, and the result showed that species at lowlands are phylogenetically clustered. The former showed similar results to the previous study on the tropical Asia (Malesia) that the phylogenetic structure of angiosperm is overdispersion at high elevations (Culmsee, Leuschner, and Richardson 2013), and the latter result have similar patterns with the Mt. Changbai (Qian, Hao, and Zhang 2014). This phenomenon is attributed to the influence of local factors, including environmental conditions and biological interactions, as well as regional processes such as geological history and speciation (Zobel 2001), although the amount of research found that the angiosperm's phylogenetic relatedness has an increasing tendency along the latitudinal gradient in China (Gheyret et al. 2020; Zu et al. 2023). However, in this study, phylogenetic relatedness did not show a significant trend along the altitudinal gradient in the subtropical mountains. These results suggest that we should focus on the effect of species' evolutionary history on the community when treating elevation and latitude as equivalent in macro‐ecological studies.

Previous study indicated that phylogenetical structure is related to taxonomic scale, and the phylogenetical structure tends to be more clustered as the scale of taxa increases (Vamosi et al. 2009). However, this study did not find a significant relationship between the NRI and the species numbers in some other mountains except Mt. Lushan. In Mt. Lushan, our study concludes that the species community in the lowland with more species and the phylogenetic structure of the species tend to be over dispersed. This might be due to the fact that the lower elevation regions of Mt. Lushan have a larger species bank, and the forest species in different succession stages are not more closely related to each other. Letcher (2009) studied the phylogenetic structure of plant communities at different stages of succession and found that with the deepening of succession, the phylogenetic structure tends to be more over dispersed (Letcher 2009).

### 4.3 How to Protect the Species Diversity in Subtropical Mountains

To reverse the trend of biodiversity loss and ensure the recovery of threatened species, the Kunming‐Montreal Global Biodiversity Framework has set a new conservation target (CBD, 2022) and outlined ambitious goals and targets for the coming decades (Antonelli et al. 2023). Under the current policy, our results provide evidence for biodiversity conservation in the subtropical mountains of China. Because biodiversity loss not only affects ecosystem functioning and the services it provides to humans (Weiser et al. 2017), but also influences the global trade and social fabric (Boonman et al. 2024). Understanding the distribution pattern of biodiversity in the mountains regions could help to assess the risk of biodiversity loss. The biodiversity loss is not only a reduction in the number of species, but also a loss of species genes, including the evolutionary history. Previous studies have concluded that biodiversity conservation should take evolutionary processes as a priority, and the conservation of phylogenetic diversity help to increase the possibility of the future diversification (Li et al. 2015; Zu et al. 2019). Along this line, we should pay attention to the habitats with high phylogenetic diversity in the elevation gradients of subtropical mountains. On the one hand, Mt. Lushan, Mt. Guanshan, Mt. Huanggang, and Mt. Fanjing should be focused on the low altitude regions. Lowlands with high plant diversity and phylogenetic diversity should be given priority protection to prevent the human influence on plant diversity in these mountains. On the contrary, the elevation distribution of plant diversity in Mt. Shennong, Mt. Jinfo, Mt. Jinggang, and Mt. Maoer shows a unimodal pattern and the peak diversity is different. If the management goal is to conserve high biodiversity, we recommend paying more attention to the higher elevations. Therefore, the conservation measures of different mountains should not be generalized, but should be focused on protection according to the difference in the elevational distribution pattern of plant diversity in different regions.

At present, despite numerous studies indicate that human disturbance is predominantly concentrated in lowland regions (Koh, Lee, and Lin 2006), the future intensification of human activities, particularly in tourism ventures such as skiing and grass skiing, which are increasingly inclined toward development in mountainous regions. This burgeoning phenomenon is poised to facilitate the further encroachment of invasive species into high‐altitude regions, thereby proffering an escalating number of opportunities for these non‐native entities to extend their influence into such regions (Pauchard et al. 2016). This incipient trend could potentially disrupt the indigenous plant diversity and community stability (Barni et al. 2012; Pauchard et al. 2016). Hence, the sanctity of plant habitats in the higher echelons of subtropical mountain ranges warrants substantial protection. Prospective research endeavors might pivot around elucidating the mechanisms through which these invasive species impinge upon high‐elevation communities, including the expansion dynamics of invasive species within mountain ecosystems.

## 5 Conclusions

This study examined the elevational distributions of plant species' multi‐dimensional diversity and phylogenetic structure along the elevational gradients in the eight subtropical mountains. We concluded that the subtropical mountains show two patterns along the elevational gradients, which are the monotonic decline and the hump‐shaped patterns. We also found more non‐native species in the high‐diversity regions of the eight mountains, and the resource opportunity hypothesis supports this finding. We suggested that conservation efforts in different mountains should not be generalized, but should focus on the peaks of diversity in different regions. Specifically, for the higher mountains, more attention should be paid to the structure and composition of forest communities at high altitudes. This is because the higher the relative altitude of the mountain, the higher the elevational distribution of its diversity. For the mountains with monotonic decreasing patterns (Mt. Lushan, Mt. Guanshan, Mt. Huanggang, and Mt. Fanjing), the lowland regions should be paid more attention because these regions have higher plant diversity and phylogenetic diversity, which are easily affected by human activities. Moreover, we did not find the same rule in these mountains: the plant species in the lower elevation regions show phylogenetic overdispersion. In other words, the phenomenon of phylogenetic clustering in high‐altitude regions is not widespread in subtropical mountains (except for Mt. Lushan). These results suggest that we should focus on the effect of species' evolutionary history on the community when treating altitude and latitude as equivalent in macro‐ecological research. Studying species diversity and phylogenetic structure at different scales could improve the understanding of evolutionary and ecological processes.

## Author Contributions

**Kuiling Zu:** conceptualization (equal), data curation (equal), investigation (equal), methodology (equal), writing – original draft (equal), writing – review and editing (equal). **Fusheng Chen:** conceptualization (equal), data curation (equal), investigation (equal), writing – review and editing (equal). **Chao Huang:** investigation (equal), methodology (equal), writing – review and editing (equal). **Yuanqiu Liu:** formal analysis (equal), investigation (equal), writing – review and editing (equal). **Fangchao Wang:** conceptualization (equal), data curation (equal), formal analysis (equal), methodology (equal). **Guojin Zhu:** formal analysis (equal), software (equal). **Wensheng Bu:** data curation (equal), formal analysis (equal), investigation (equal). **Xiangmin Fang:** conceptualization (equal), data curation (equal), formal analysis (equal), funding acquisition (equal), writing – review and editing (equal). **Liping Guo:** investigation (equal), methodology (equal).

## Conflicts of Interest

The authors declare no conflicts of interest.

## Supporting information


Data S1.

## Data Availability

The data that support the results of this study for the peer review are available in this article and the Supporting Information of this article. The raw data for this study are available via the Dryad Digital Repository upon acceptance of the manuscript (https://doi.org/10.5061/dryad.rn8pk0pmr). Reviewer sharing link is: http://datadryad.org/stash/share/wsTLGbCO4Ze32mNKeCK‐JFYSavGWVYWtXM4g0zn9SUo. All the data are useful for the peer review process.
