# Supplementary material for: The Elevational Distribution Patterns of Plant Diversity and Phylogenetic Structure Vary Geographically Across Eight Subtropical Mountains
Source: Ecol Evol. 2024 Dec 17;14(12):e70722. doi: 10.1002/ece3.70722 (PMC11652111; doi:10.1002/ece3.70722)
Supplement: Supplementary file 1 — Data S1. [file ECE3-14-e70722-s001.docx]

**The elevational distribution patterns of plant diversity and phylogenetic structure vary geographically across eight subtropical mountains**

**Supporting information:**

**Table S1: Plant distribution data from the literature and books for each mountain.**

Duan, W.J., Wang, J.Y., 2013. Vertical distribution pattern and determinant analysis of forest community in Maoer Mountain National Nature Reserve. Ecol. Environ. Sci. 22(4), 563-566.

Huang, X.Y., Xie, Q., Ding, L.Q., et al., 2023. Floristic Characteristics and Community Succession in Alpine Wet-land of Mao’er Mountain, Guangxi, China. Chinese Journal of Tropical Crop 44 (04), 846-857.

Li, L., Wei, S.G., Lian, J.Y., Cao, H.L., et al., 2020. Distributional regularity of species diversity in plant community at different latitudes in subtropics. Acta Ecol. Sin. 40(4), 1249-1257.

Liao, W.B., Wang, Y.Y., Li, Z., et al.,2014. Comprehensive Scientific Expedition on Biodiversity in Jinggang Mountains, China, Beijing: Science Press.

Liu, X.Z., Wang, L., et al., 2010. Examination and Research on Biodiversity in Mount Lushan Nature Reserve, Jiangxi Province, China. Beijing: Science Press.

Song, Y.C., 2013. Chinese evergreen broad-leaved forest. Beijing: Science Press.

Tan, Y.B., Fu, Z., Tian, H.D., et al., 2019. Factors Influencing Herbaceous Plant Height in Forest Communities at Different Altitudes in Mao'er Mountain. Guangxi Forestry Science 2019(4), 472-478.

Wu, S.Y., Cheng, F.B., Guo, Y.R., 2018. Inventorying Plant Diversity in Wuyi Mountain National Nature Reserve, Jiangxi, China. Hong Xing Electronic Audio& Visual Press.

Zhu, B., Chen, A.P., Liu, Z.L., et al., 2004. Changes in floristic composition, community structure, and tree species diversity of plant communities along altitudinal gradients on Mt. Mao’er, Guangxi, China. Biodivers. Sci. 12(1), 44.


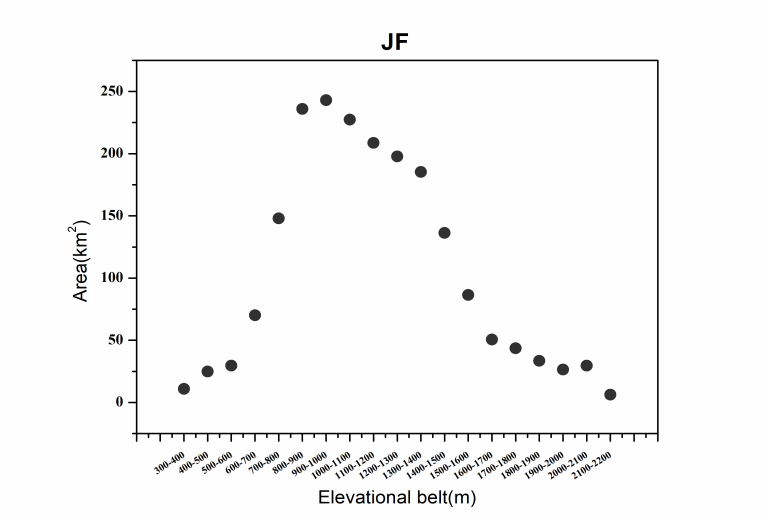

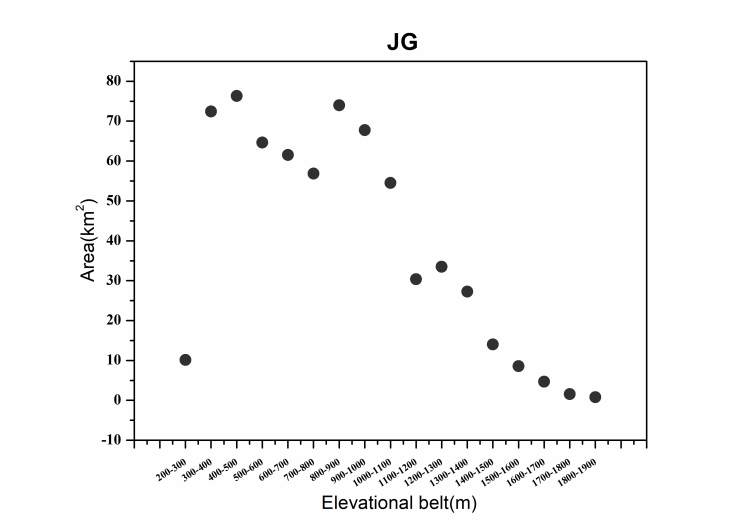

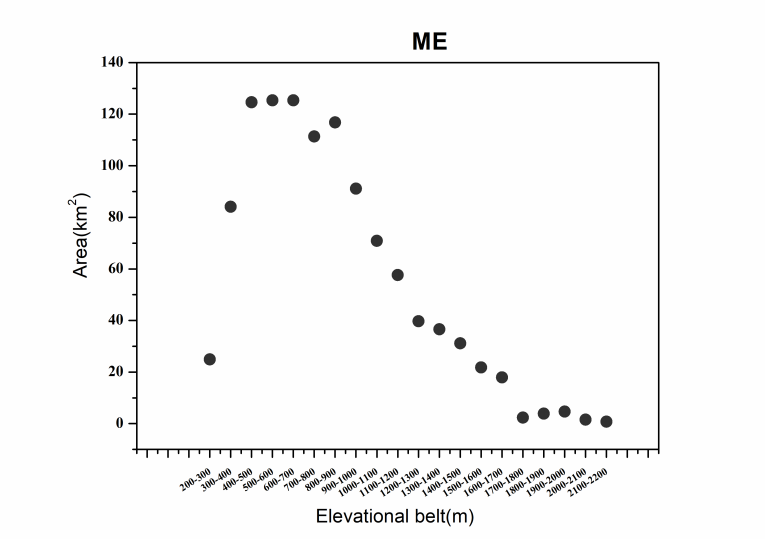

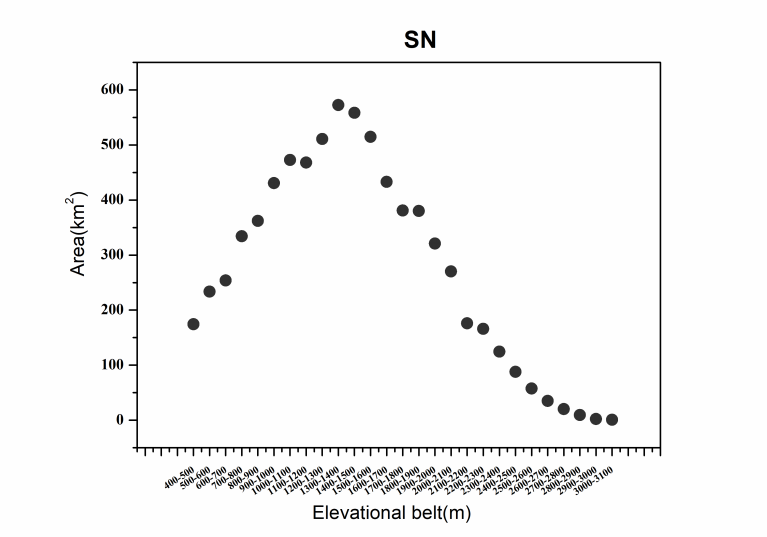

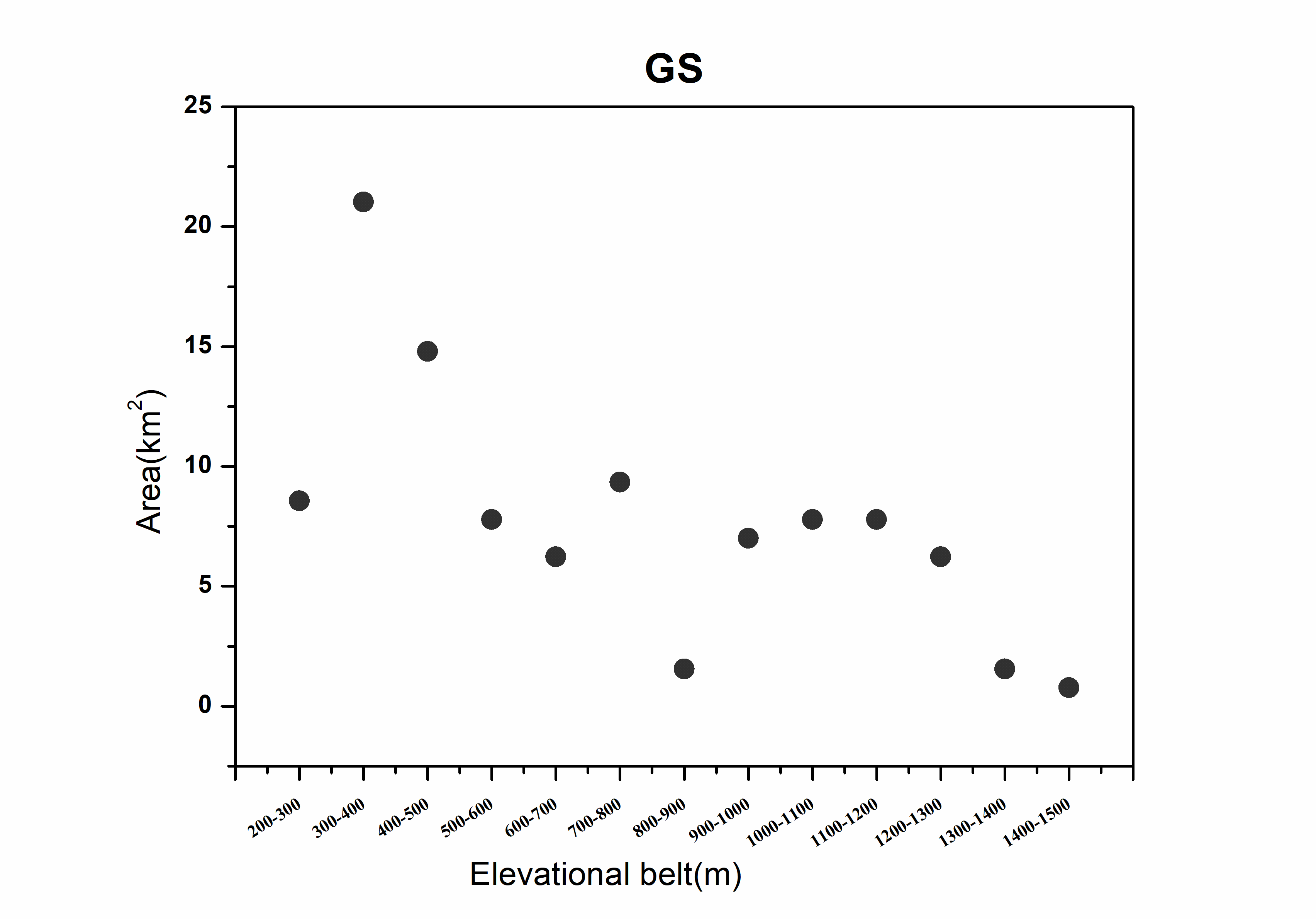

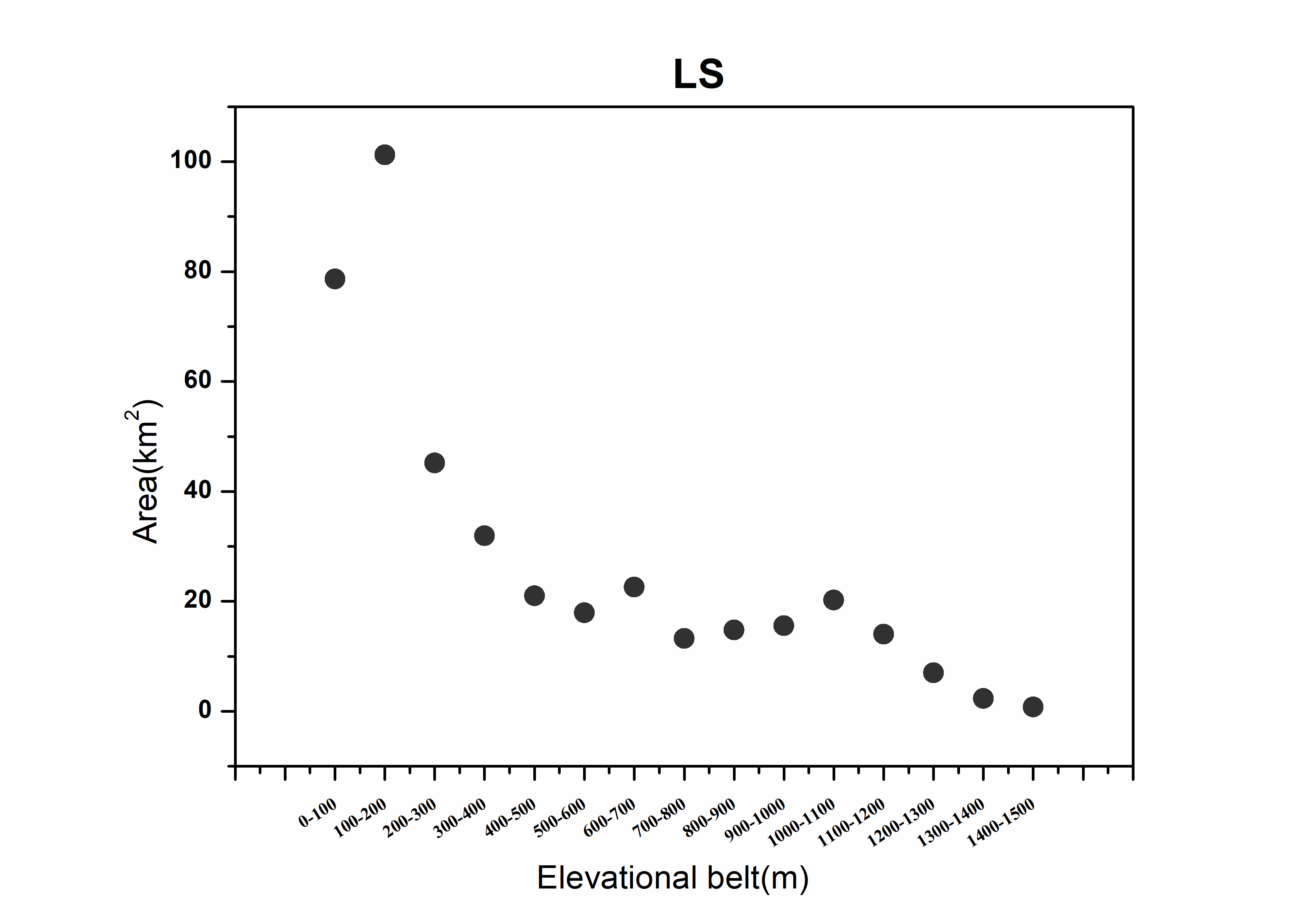

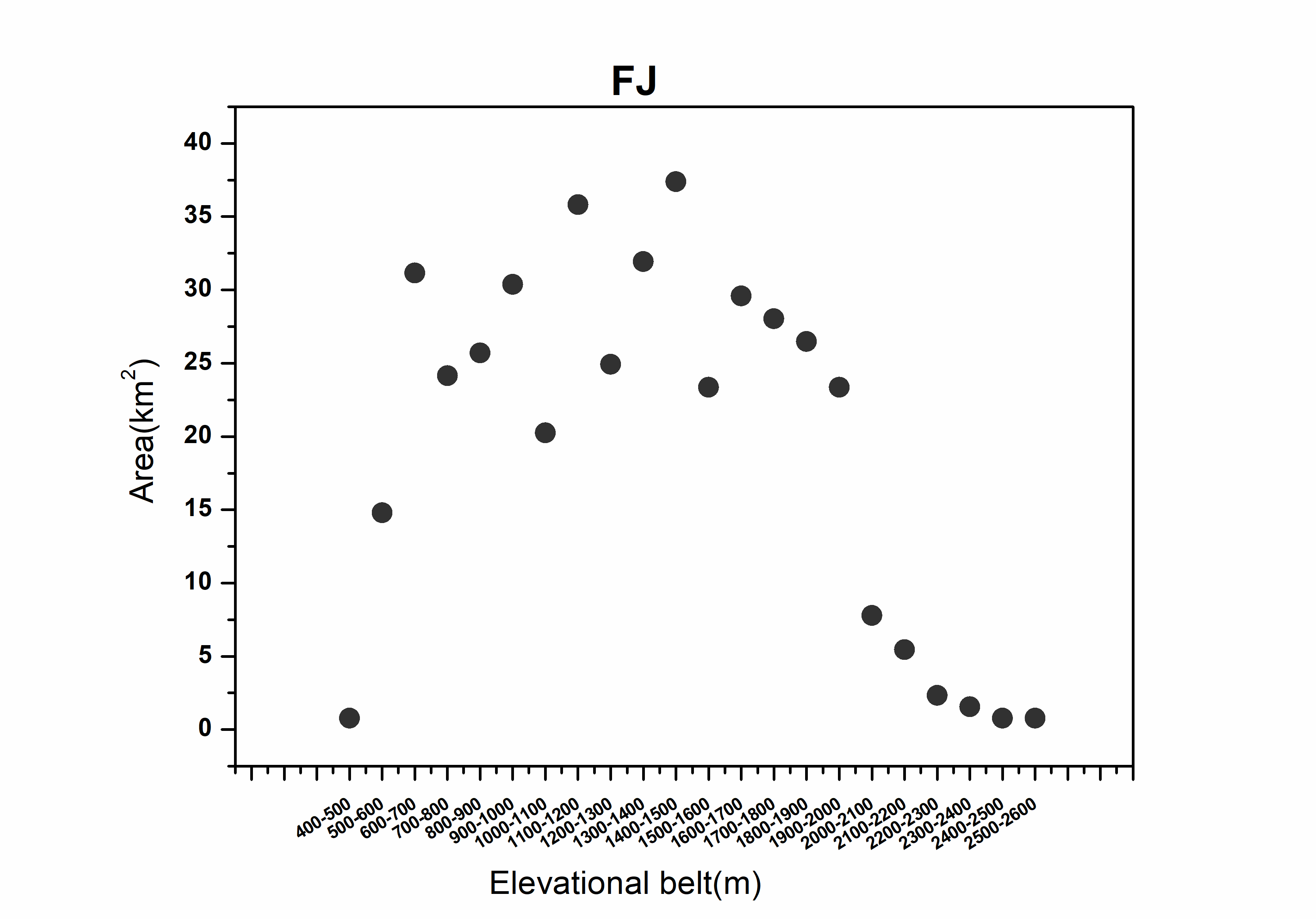

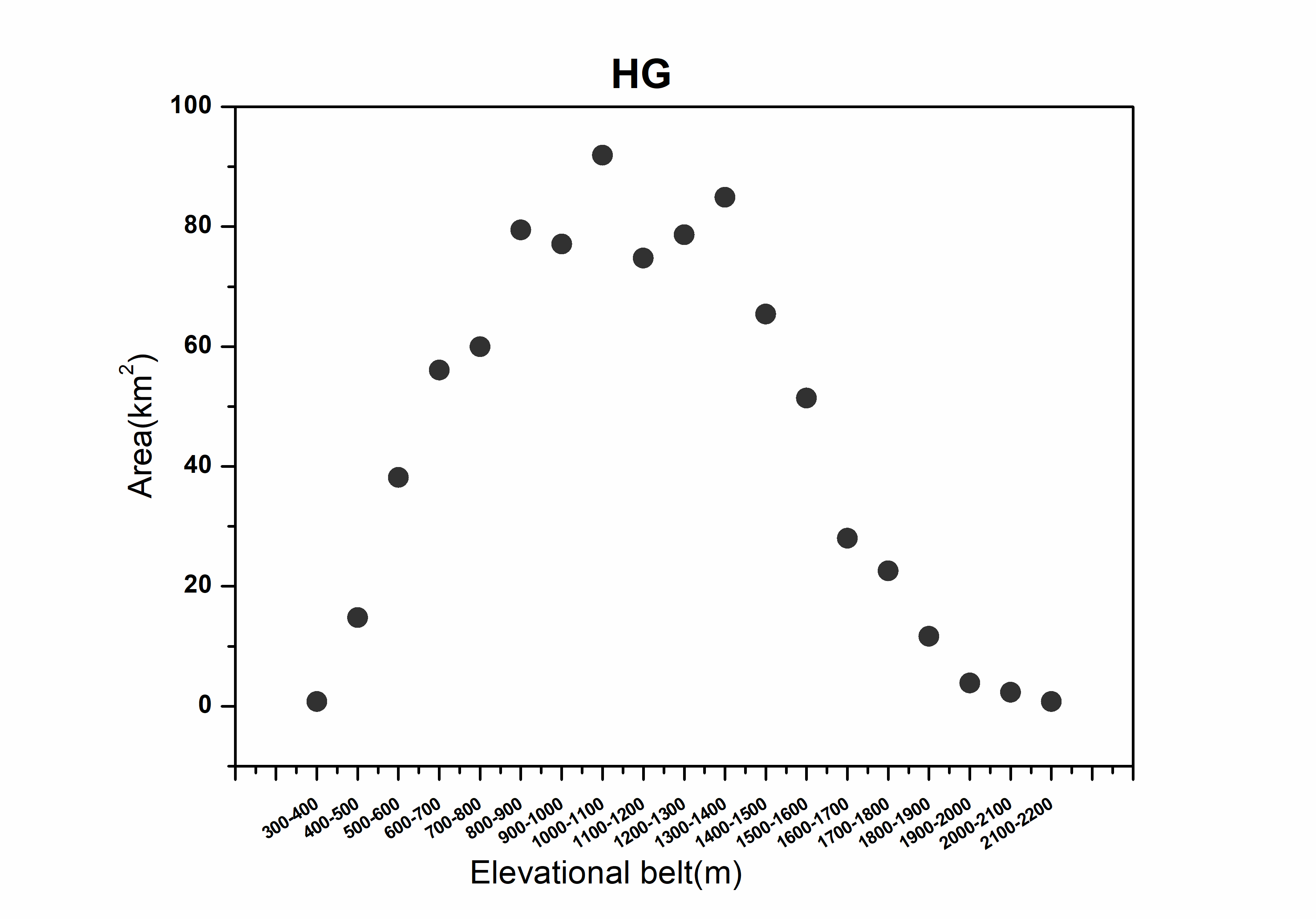


**Fig. S1. The distributional area of different mountains along the elevational gradients.**

**Table S2 The number of non-native species of the eight mountains.**

| Mount | Abbr. | Species | Genus | Family |
| --- | --- | --- | --- | --- |
| Guanshan | GS | 35 | 29 | 20 |
| Lushan | LS | 207 | 153 | 62 |
| Fanjing | FJ | 26 | 26 | 16 |
| Huanggang | HG | 57 | 53 | 32 |
| Shennongjia | SNJ | 152 | 116 | 48 |
| Jinfo | JF | 129 | 103 | 45 |
| Jinggang | JG | 49 | 41 | 22 |
| Maoer | ME | 59 | 51 | 28 |
